# Double blind microarray-based polysaccharide profiling enables parallel identification of uncharacterized polysaccharides and carbohydrate-binding proteins with unknown specificities

**DOI:** 10.1038/s41598-018-20605-9

**Published:** 2018-02-06

**Authors:** Armando A. Salmeán, Alexia Guillouzo, Delphine Duffieux, Murielle Jam, Maria Matard-Mann, Robert Larocque, Henriette L. Pedersen, Gurvan Michel, Mirjam Czjzek, William G. T. Willats, Cécile Hervé

**Affiliations:** 1 Department of Plant and Environmental Sciences, University of Copenhagen, Thorvaldsensvej 40, 1871 Frederiksberg, Denmark; 2 Sorbonne Universités, UPMC Univ Paris 06, CNRS, UMR 8227, Integrative Biology of Marine Models, Station Biologique de Roscoff, CS 90074 Roscoff, France; 3 Present Address: William G.T. Willats, Newcastle University, Newcastle upon Tyne, United Kingdom

## Abstract

Marine algae are one of the largest sources of carbon on the planet. The microbial degradation of algal polysaccharides to their constitutive sugars is a cornerstone in the global carbon cycle in oceans. Marine polysaccharides are highly complex and heterogeneous, and poorly understood. This is also true for marine microbial proteins that specifically degrade these substrates and when characterized, they are frequently ascribed to new protein families. Marine (meta)genomic datasets contain large numbers of genes with functions putatively assigned to carbohydrate processing, but for which empirical biochemical activity is lacking. There is a paucity of knowledge on both sides of this protein/carbohydrate relationship. Addressing this ‘double blind’ problem requires high throughput strategies that allow large scale screening of protein activities, and polysaccharide occurrence. Glycan microarrays, in particular the Comprehensive Microarray Polymer Profiling (CoMPP) method, are powerful in screening large collections of glycans and we described the integration of this technology to a medium throughput protein expression system focused on marine genes. This methodology (Double Blind CoMPP or DB-CoMPP) enables us to characterize novel polysaccharide-binding proteins and to relate their ligands to algal clades. This data further indicate the potential of the DB-CoMPP technique to accommodate samples of all biological sources.

## Introduction

The coevolution of marine macro-algae and heterotrophic microbes underpins marine ecosystem development and has a major impact on global carbon cycling. This coevolution is defined on one side by immensely complex and heterogeneous algal biomass, and on the other by a correspondingly complex set of microbial processing proteins. Both sides are poorly understood. The cell walls of marine macro-algae are fibre-composite materials consisting of interlinked networks of complex polysaccharides. A remarkable diversity of cell wall polymers has evolved to meet a wide range of developmental roles. Diversity is generated by biosynthesis and post-synthetic modifications to enable fine-tuning matched to local functional requirements. Although constructed on similar principles to the cell walls of land plants, they differ significantly because of an abundance of uronic and sulfated polysaccharides^1^.

Algal cell wall polysaccharides are an important nutrient source for marine microbes, and polysaccharide complexity and diversity is matched by a large number of microbial modular carbohydrate active enzymes (CAZymes) and binding modules (CBMs), tailored to specifically hydrolyse and recognize the unique sugar-units of these glycans^2^. CAZymes and the associated carbohydrate binding modules (CBMs) are classified into sequence-based families in the CAZy database (www.cazy.org/)^3^. It is noteworthy that marine enzymes and modules often represent novel families or are present as additional sub-families within those of the glycoside hydrolase CAZy classification^2^. Indeed, the current *status quo* is that genomic and metagenomic data from marine environments is most often annotated with biochemical knowledge derived almost entirely from bacteria and fungi that decompose terrestrial plants. However, marine primary producers and land plants share only a limited number of polysaccharides, for example starch, cellulose and certain mixed linkage glucans^4–6^. While cellulose dominates terrestrial biomass, it is far less abundant in marine environments where it is typically only present as minor component of macroalgal cell walls.

Marine heterotrophic bacteria (MHB) have become specialized to exploit the abundant carbon source of macro-algal biomass. Recent studies about diversity and abundance of MHB in the marine environment have revealed the major role of some groups as carbon recyclers, which are mainly *Alpha-* and *Gammaproteobacteria*, *Bacteroidetes* or *Planctomycetes*^7–12^. In this context, the flavobacterium *Zobellia galactanivorans*, a member of the *Bacteroidetes* group, is now well established as a model marine carbohydrate degrader^13^. Characteristic of *Bacteroidetes*, the components for efficient carbohydrate degradation, such as CAZymes, sulfatases, transporters and regulators are mainly clustered into Polysaccharide Utilization Loci (PULs)^14^. Fifty such carbohydrate degrading systems, organized in PULs, were identified in *Z. galactanivorans*^13^ (see Supplementary Table S3 of this reference). By definition, the hallmark of a PUL is the presence of a TonB dependent receptor (TBDR) gene followed (or preceded) by a so-called *susD*-like gene^15^. The latter are named after the first such protein to be identified in the gut bacterium *Bacteroides thetaiotaomicron*, the ‘starch utilizing system protein D’ (SusD), which is involved in the sensing and uptake of starch. The structure and function of SusD was extensively studied^14^ and was found, together with SusC (the corresponding TBDR), to be required for starch recognition, binding and uptake^16^ in *B. thetaiotaomicron*. Analogous PUL systems have since then been extensively studied^17,18^, albeit to date with a focus on those active on polysaccharides of the terrestrial environment.

The degradative processes underlying the decomposition of algal biomass is of considerable ecological and biotechnological importance but the mechanistic details of these processes are largely obscure at present. Although a wealth of metagenomic data is available, the empirical characterization of large numbers of predicted proteins, enzymes and modules remains a major challenge^19,20^. We currently lack knowledge about both the precise nature of the algal biomass, and the microbial proteins. In this work we tackled this ‘double blind’ problem by exploiting the high-throughput capacity of carbohydrate microarray technology, in particular the Comprehensive Microarray Polymer Profiling (CoMPP) method, which has been successfully applied to streptophyte systems^21,22^. Multiplexing in the microarray platform was supported by a medium throughput protein expression system. We used this integrated platform that we called Double Blind CoMPP (DB-CoMPP) to characterize novel marine CBMs and SusDs and to map ligand recognition to distinct algal clades. In so doing we provide new insight into the binding and substrate specificities of some marine CBMs and SusD-like proteins, as well as the presence of peculiar polysaccharide epitopes in marine macro-algal cell walls.

## Results

### Development of a double blind microarray-based strategy for glycome analysis

**Figure 1:**
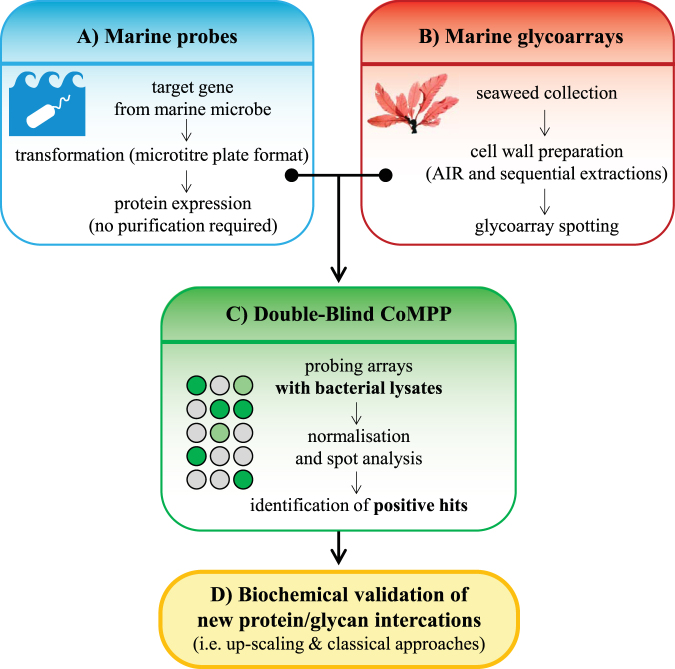
Schematic of the DB-CoMPP technique. An example using marine samples is shown. (**A**) Genes encoding carbohydrate-binding proteins with unknown specificities are selected and the corresponding recombinant proteins are expressed in a medium throughput manner. (**B**) Cell wall polymers are sequentially extracted using dedicated protocols. They are printed onto the array following the standard CoMPP technique. (**C**) The arrays are probed with the supernatants of bacterial lysates. Signals from negative controls are used to subtract background. The samples giving positive signals are identified. (**D**) The purified probes and their corresponding ligands are gathered in larger amounts and used for biochemical validation of protein-glycan interactions.

**Figure 2:**
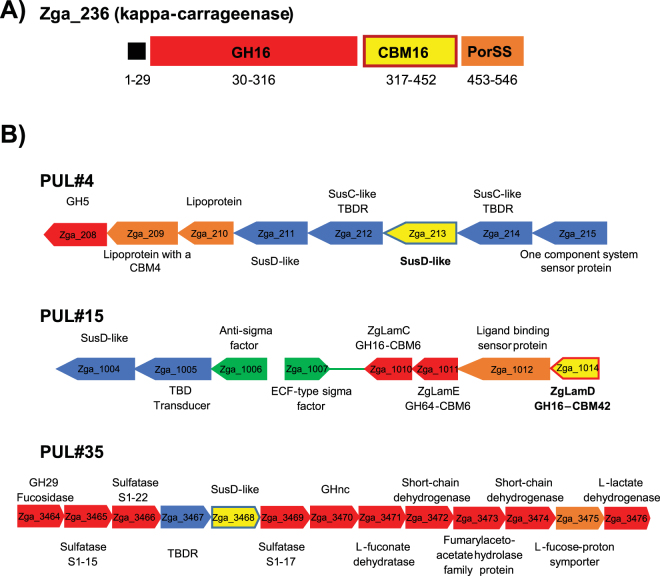
Gene contexts of four selected protein-targets from *Zobellia galactanivorans*. (**A**) Modular architecture of the kappa-carrageenase CgkA (Zga_236). (**B**) Genetic context of SusD-213, CBM42 and SusD-3468 in PULs.

The DB-CoMPP technique combines the use of two high-throughput systems; one for protein expression and one for glycan screening (Fig. 1) and its strategy can be divided into four main stages: a) heterologous expression of the targeted proteins in a high throughput manner, b) preparation of microarrays using standard CoMPP and with a dedicated multi-step extraction regime, c) probing the arrays with the supernatants of the bacterial lysates and d) validation of the most promising targets. In this work we were interested in discovering original glycan structures and/or novel carbohydrate-binding proteins originated from a marine environment. We heterologously expressed carbohydrate binding proteins from *Z. galactanivorans*, which is a model marine bacterium for the bioconversion of algal polysaccharides. The targeted proteins encompass carbohydrate binding modules (CBMs) and SusD-like proteins, all identified during the annotation of the genome sequence of *Z. galactinovorans*^13^. As an illustration, the detailed gene contexts of four of those (i.e. catalytic domain with an appended CBM or clustering in PULs), are illustrated in Fig. 2. All targets are listed in Supplementary Table S1. The proteins were expressed in *E. coli* via a medium throughput system based on a microtiter plate format and which includes two expression plasmids encoding either a His- or GST-tag^19^. For each target, the tag version giving the highest expression yield in term of soluble protein, was selected for further study. In parallel of the expression procedure we prepared CoMPP arrays using cell walls from algae of diverse types and land plants. The cell walls were sequentially extracted using dedicated protocols for each of the three constitutive groups (i.e. brown algae, red algae and green algae/plants) in order to solubilize and separate major classes of cell-wall polysaccharides. A variety of defined commercial polysaccharides were also included as internal standards. These marine glycoarrays were probed following the standard CoMPP method^23^.

### Sensitivity, repeatability and time-cost optimization of the DB-CoMPP technique

While protein expression can be done in a medium-throughput manner, conventional techniques for biochemical characterization of proteins still necessitate scaling up and purification. These last procedures are time-consuming and labour intensive, since each protein is usually studied on an individual basis. However, if a carbohydrate-binding protein has a strong avidity for its target ligands, the purification stages are optional with the use of glycan arrays, as the washing steps move away all unbound proteins. We take advantage of this property and probed the CoMPP arrays directly with the supernatants of the bacterial lysates (Fig. 1), therefore greatly increasing throughput in the identification of new proteins of interest. The repeatability of the DB-CoMPP technique was tested by comparing data derived from three independent protein productions. To increase the robustness in signal assignment, the extracts are usually printed with repetitive spot frequencies on CoMPP arrays^21,22^ and the same strategy was applied for the DB-CoMPP technique (16-spot sub-array per sample). If a DB-CoMPP regime similar to that described in Fig. 1 is used, up to 96 proteins can be produced and screened on a 100 extracts-based array, and analysed in two weeks.

### DB-CoMPP analysis of marine samples

**Figure 3:**
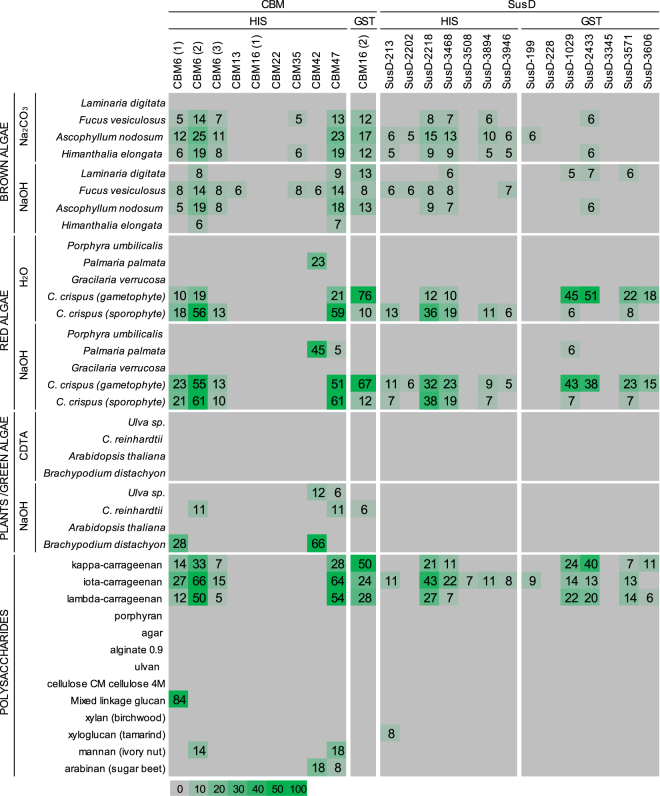
DB-CoMPP analysis of marine samples. CoMPP binding profiles of the supernatants of bacterial lysates towards a range of cell wall extracts and commercial polysaccharides. Results are means of three individual experimental replicates. The colour scale in relation to absorbance values is shown. The highest mean signal value in the entire data set was set to 100 and all other signals adjusted accordingly. Values < 5 were considered as background and discarded.

In order to assess the specificity of the method to report glycan recognition, an *E. coli* strain expressing the characterized CBM6 from *Z. galactanivorans* and that binds (1,3)-β- and (1,3),(1,4)-β-D-glucans^24^ was included as a positive control and referred as CBM6(1). Negative controls were prepared in the same way and comprised pure *E. coli* strains (no plasmids), *E. coli* strains expressing simple plasmid insertions (plasmids with no heterologous gene) and *E. coli* strains expressing proteins with no glycan binding ability (B-PAB0034 previously published^19^; Supplementary Table S1). In the present study 65% of the targets were expressed as soluble proteins, which is in accordance with previous results obtained for marine bacterial genes including genes from *Z. galactanivorans*^19^. The mean values for the binding of the bacterial lysates to the marine glycoarrays are shown as heatmaps, where spot signals are correlated to colour intensity. Some of the negative controls produced weak signals which was defined as unspecific binding (Supplementary Fig. S1). These values were subtracted from the initial dataset (Supplementary Fig. S2) to give the final heatmap shown in Fig. 3 and from which positive protein-glycan interactions and signal intensities, as an indication of relative avidities, can be inferred. The highest mean signal value in the entire data set was set to 100 and all other signals adjusted accordingly The bacterial lysates gave positive signals with intensities evenly distributed within the 0–100 interval, and this is visually comparable to results obtained with standard CoMPP^21,23^. Positive signals were observed independently of the protein type (CBM or SusD) and of the nature of the tag (His or GTS) involved in the detection process. There was a general tendency towards binding to carrageenans and cell wall extracts from carrageenophytes as illustrated by *Chondrus crispus* (Fig. 3). The strong electrostatic properties of carrageenans might lead to unspecific binding^25^. Although some of the expressed proteins might be specific for carrageenans, the signals originated from these polysaccharides were considered with great caution at this stage. As expected, the binding profile of CBM6(1)was in good agreement with its published specificities^24^, with a strong binding toward mixed-linkage (1,3),(1,4)-β-D-glucan (MLG). The polysaccharide was successfully detected in *Brachypodium distachyon*, which is known to have the highest content of MLG among grasses^26^. No signal was detected with *Arabidospis thaliana* which does not contain MLG.

One of the primary aims in developing the DB-CoMPP technique was to set up a method that allows a rapid screening of a collection of carbohydrate-binding proteins with unknown specificities towards a large variety of dedicated glycan samples. As proof of concept, four targets were selected for further study, namely CBM16(2), CBM42, SusD-213 and SusD-3468. The rationale to choose these proteins was to challenge the binding abilities predicted from the gene contexts (when predicted; see Fig. 2 and Supplementary Table S1) to the ones reported by the DB-CoMPP profiles (Fig. 3). CBM16(2) and CBM42 gave positive signals with marine polysaccharides. On glycoarrays CBM16(2) bound strongly to carrageenans. In its natural context the module is appended to a κ-carrageenase which strengthened the assumption of a carrageenan-binder. The binding profile for CBM42 was very distinct as compared to the other probes, with a consistent binding of all *Palmaria palmata* extracts (Fig. 3), which cell walls contain almost exclusively mixed-linkage (1,3),(1,4)-β-xylan (MLX)^27^. Additional binding of this cell lysate was observed towards *B. distachyon* and arabinan. CBM42 is naturally attached to a laminarinase^28^ and one would expect this probe to bind (1,3)-β-glucans in first instance. The binding of the bacterial lysate containing SusD-213 was also peculiar in binding xyloglucan, a plant cell-wall polysaccharide unknown to be present in red or brown algae. No putative ligand could be inferred for SusD-3468 by the DB-CoMPP analysis alone and it is possible that the background signal with carrageenans interfered with real ligand recognition. An additional reason for this selection included the fact that the probes were easily expressed as soluble material (Supplementary Fig. S3). The two distinct tags were represented among the probes used, which was also seen as an interest to assay two detection methods.

### Validation of selected binding abilities by standard CoMPP and microtiter plate assays

**Figure 4:**
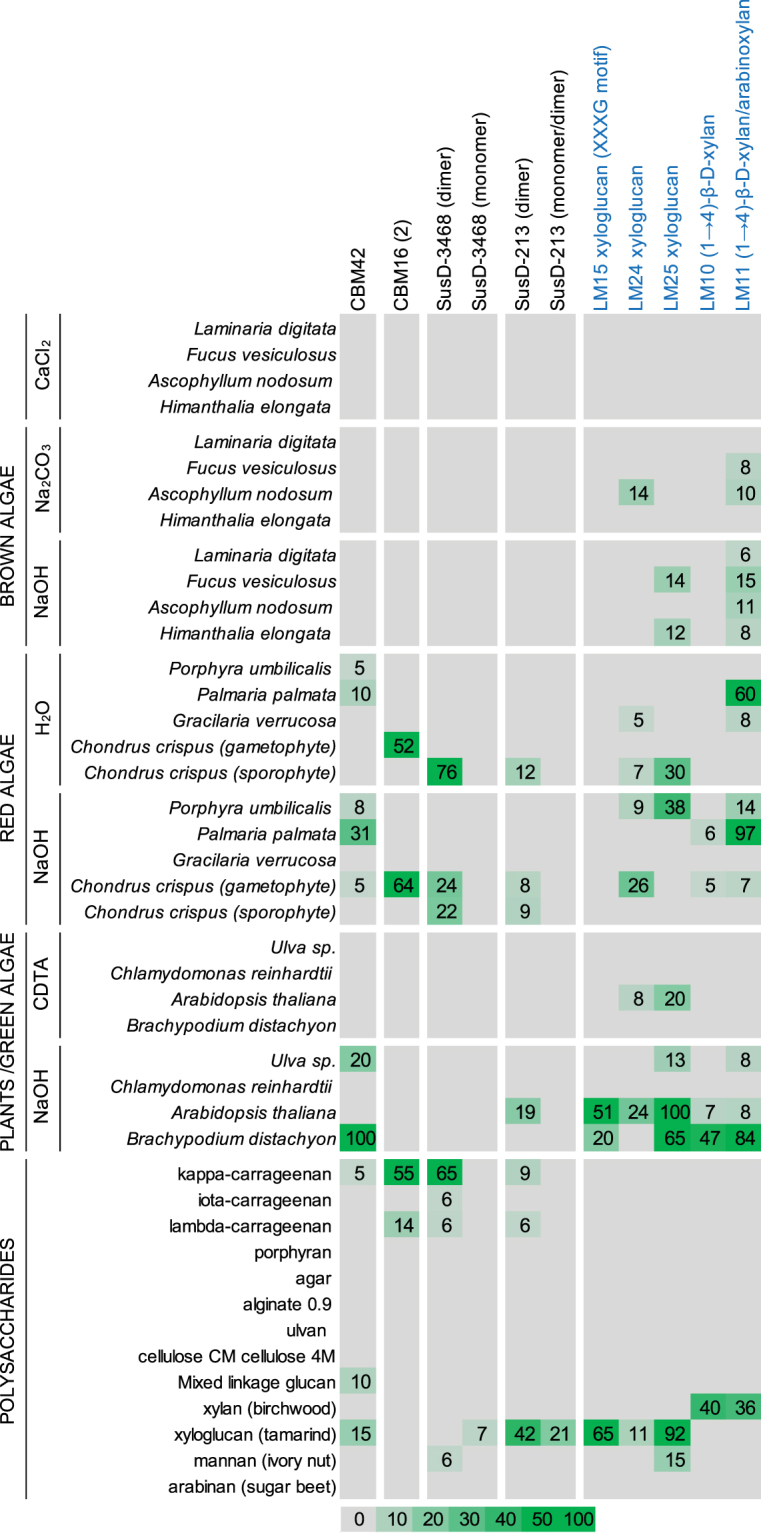
Evaluation of the recognition abilities of the purified probes by standard CoMPP analysis. CoMPP binding profiles of purified CBM and SusD-like proteins towards a range of cell wall extracts and commercial polysaccharides. Monoclonal antibodies (LM10, LM11, LM15, LM24, LM25) that bind the indicated plant polysaccharides were used as binding controls. Results are means of three individual experimental replicates. The colour scale in relation to absorbance values is shown. The highest mean signal value in the entire data set was set to 100 and all other signals adjusted accordingly. Values < 5 were considered as background and discarded.

**Figure 5:**
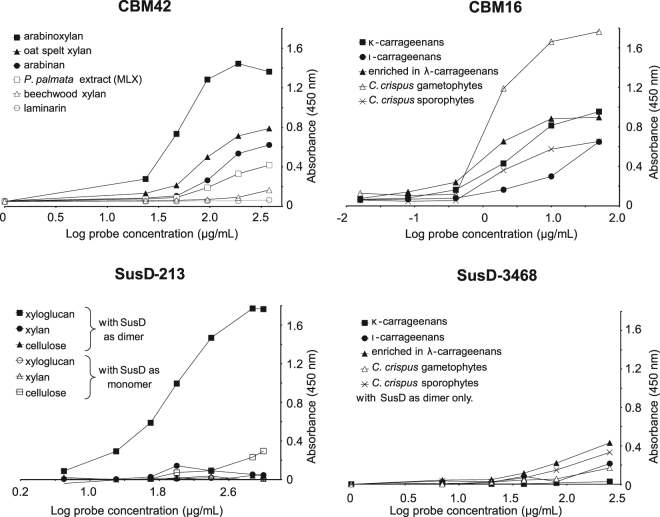
Biochemical validation of the binding abilities of the purified probes by microtiter plate assays. Binding of CBM42, CBM16(2), SusD-213 and SusD-3468 in capture microtiter plate assays with a selection of purified polysaccharides and cell wall extracts as immobilized ligands.

The expression volumes were scaled up for CBM16(2), CBM42, SusD-213 and SusD-3468, and the probes purified. Gel-filtration chromatography analysis of the two SusD-like proteins indicated that they were both naturally exhibiting dimeric and monomeric forms (Supplementary Fig. S4). The six purified samples were screened on CoMPP arrays and their binding abilities compared with monoclonal antibodies of known specificities (Fig. 4). The bindings of the probes were additionally investigated by microtiter plate assays by using the most effective ligands determined by CoMPP, with the addition of related polymers (Fig. 4). The microtiter plate assay is an easily-handle technique, initially used for antibodies but extended toward CBMs, for the screening of recognition specificities^29^. It offers the possibility to rapidly validate (or invalidate) the DB-CoMPP results. The binding abilities as observed by the DB-CoMPP technique were confirmed for all probes and the targeted ligands more precisely defined. CBM42 showed recognition of *P. palmata* extracts (Fig. 3) and a screening with xylan-related polymers indicated that it binds preferentially arabinoxylan (Fig. 5 and Supplementary Fig. S5). LM10 and LM11 both recognize unsubstituted (1,4)-β-xylans, whereas LM11 can also bind to arabinoxylan^30^. As a control for the CBM42 binding profile, LM11 but not LM10 displayed binding to all *P. palmata* extracts (Fig. 4). CBM16(2) showed extensive binding abilities towards carrageenans, with a preference towards κ-carrageenans. NMR analyses indicated that the commercial λ-carrageenan samples also contained κ- and ι-forms (data not shown), we therefore produced in-house cell wall extracts from *C. crispus* by separating the life-stages as a starting point (gametophytes only produced κ-/ι-carrageenans and sporophytes only produced λ-carrageenans)^31^. The results obtained indicated that CBM16(2) preferentially binds κ-carrageenans (Figs 3 and 4). In the case of the SusD-like proteins, the dimers were much more effective to bind ligands than the momoners (Figs 4 and 5). The ability of SusD-213 to bind xyloglucan was clearly confirmed (Fig. 5). The three antibodies used to control xyloglucan recognition (LM15, LM24, LM25), have been previously shown to display differing binding profiles toward xyloglucan-derived oligosaccharides^22^. In particular both LM24 and LM25 display wider recognition of galactosylated xyloglucan oligomers than LM15, with LM25 being able to recognise additional β-glucan epitopes^22^. While the recognition profile of SusD-213 (Fig. 4) is indicating subtle differences in its binding specificity as compared to the antibodies, it is somewhat closer to the binding profile of LM24. This observation is possibly indicating a preference toward galactosylated xyloglucan epitopes. The SusD-3468 showed strong recognition of carrageenans on the arrays (Fig. 4). However there is a discrepancy in the results between the commercial and extracted ligands with the recognition of either λ- or κ-/ι-carrageenans, respectively. The microtiter plate assay did not reveal any major binding towards these substrates (Fig. 5).

## Discussion

We have implemented a combined high-throughput protein expression strategy with comprehensive profiling using an algal glycan-microarray that we call DB-CoMPP. This approach enables the rapid screening of a collection of bacterial strains expressing carbohydrate-binding proteins against dedicated known and unknown marine glycan samples. In this procedure, the ability to survey the binding ability of recombinant proteins expressed in minute volumes and without scaling up the purification step, enhances the chances to identify new carbohydrate-binding proteins and/or to reveal new information about the occurrence of specific carbohydrate components in samples. We present here the application of this DB-CoMPP to marine predicted proteins and polymer samples and their validation, but the method can readily be adapted to samples of any origin, with additional or different extraction regimes, and additional glycan probes.

The DB-CoMPP technique applied on our marine samples provided several positive signals apparent from algal extracts. It shows the detection of known polysaccharides in new biological contexts, with notably the identification of arabinoxylan and xyloglucan-related epitopes in red algae. As compared to land plants, the current knowledge on the cell wall as a comprehensive structure in red seaweed is limited. The gelling and thickening water-soluble galactans in red algal cell walls have been described in some details (i.e. carrageenans, agars, porphyrans^32,33^), but very little is known of the nature of additional neutral and/or crystalline polysaccharides^31,34^. They usually represent a small portion of the wall (1–8% of algal dry weight), with the exception of *Palmaria palmata* which does not produce matrix galactans, but almost exclusively MLX with minute amounts of cellulose and (1,4)-β-xylans^27^. Using a similar initial glycoarray screening, the existence of (1,3)-β-glucans^35^ and water-insoluble MLG^6^ has been recently shown and validated in brown algal cell walls^4^. Altogether these results indicate that novel detailed investigations of glycan structures are needed for algal cell walls, and one can argue that it might lead to the revision and/or the establishment of detailed cell wall compositions for some species. The use of a marine bacterium was instrumental in the present study to identify new marine polysaccharides, as this model organism is naturally adapted to catabolize these polymers^13^. These organisms also offer additional sources of carbohydrate-binding probes, as compared to the ones classically used in plant cell wall studies, to investigate cell wall structures, functions and dynamics. As an example, the genome of *Z. galactanivorans*, a heterotrophic algal associated marine *Flavobacteriia*, contains 72 SusD-like proteins organized in 50 distinct carbohydrate degrading PUL, 37 CBMs covering 14 different CBM families, not mentioning the numerous modules classified as unknown (UNK) or X modules that might be novel CBMs^13^.

Thus, at the same time as identifying polysaccharides, the DB-CoMPP technique allowed us to identify novel binding probes, specific of marine polysaccharides such as κ-carrageenan, for which no CBM has been identified to date. Appended to a GH16 catalytic module that is annotated as a κ-carrageenase (Fig. 2), the true biochemical binding affinity of the CBM16(2) analysed here still needs to be determined. Indeed, the demonstrated binding properties reported to date on CBMs from family 16 are β-1,4-linked glucose containing substrates, such as cellulose or glucomannan^36^ and these polysaccharide structures are far from resembling that of κ-carrageenan. Our method thus allowed us, for the first time, to identify and characterize a κ-carrageenan binding CBM module. More surprisingly, the binding properties for CBM42, revealed by the DB-CoMPP and subsequent in-depth characterization, were to be specific of arabinoxylan. Albeit the fact that previously characterized CBM42s have been shown to be specific of arabinofuranose^37^, the presence of this module attached to a GH16 catalytic domain annotated as a laminarinase^28^ (Fig. 2) was not at all indicative of such a binding specificity. In addition, the occurrence of arabinoxylan in cell walls of marine algae has not been described to date either. Consequently, the characterized binding specificity of this marine CBM42 not only points towards the presence of unsuspected arabinofuranose-branching of xylan polysaccharides in seaweed cell walls, but also questions the function of the appended GH16 catalytic module (Fig. 2). Based on sequence comparison (31% sequence identity with the characterized (1,3(4))-β-glucanase from *Rhodothermus marinus*) and phylogenetic analysis^38^, this GH16 module seems a regular (1,3(4))-β-glucanase (EC number 3.2.1.6). In this case, this catalytic module could degrade (1,3)-β-glucans or (1,3),(1,4)-β-D-glucans in a cell wall context in which arabinoxylans would also be present. Such modular CAZymes in which the catalytic module and the CBM do not target the same polysaccharide have already been observed in the context of plant cell walls, notably cellulose-binding CBMs which are often components of enzymes that hydrolyze xylans, mannans, and pectins^39^. An alternative scenario is that this GH16 could be specific for an arabinoxylan-related polysaccharide, despite its phylogenetic position within the GH16 (1,3(4))-β-glucanase subfamily.

In *Bacteroidetes* CAZymes are often found in so called Polysaccharide Utilizing Loci (referred to as PULs)^40^. These gene clusters encode complete protein systems for the degradation of polysaccharides and the uptake of the degradation products. Each PUL usually orchestrates the breakdown of a single specific glycan^40–44^ and the complexity of the glycan is reflected by the complexity of the enzymes found in the PUL^18^. This tendency has been essentially observed in human gut bacteria^40–44^, but the recent characterization of the carrageenan-specific PUL in *Z. galactanivorans*^45^ shows that it is also the case at least in some marine bacteria. Nonetheless, this is not an absolute rule since more versatile PULs have been found in reindeer rumen bacteria^46^. In this context, the presence of the modular GH16-CBM42 gene in a PUL like gene context (Fig. 2, PUL#15 from Barbeyron *et al*.^13^) suggests that the adjacent sensor-protein may also have an affinity for an arabinoxylan-related polysaccharide. Similarly, the identified xyloglucan binding properties of SusD-213 allows extrapolation of potential xyloglucan activity/specificity to the proteins encoded by the surrounding genes of the PUL gene structure #4 (Fig. 2). Therefore, the application of the DB-CoMPP method to unknown modules extracted from Bacteroidetes PULs, screened against known or unknown polysaccharides, has the other advantage to reveal additional enzymes, transporters, or sensing proteins that are present in the same PUL and which might have a common glycan target. The predictive power of CoMPP screening on modules originating from *Bacteroidetes* PUL has already been used in previous studies. For example, Mackenzie *et al*.^46^ describe the enzymatic capabilities of a PUL encoded within a numerically abundant uncultured rumen phylotype, using CoMPP on the glycan content of the rumen of grazing reindeers^46^. In our study, the additional complexity arises from the fact that not only specific antibodies or CBMs that target marine polysaccharides are rare but also the knowledge about diversity and fine structure of algal cell wall polysaccharides is to date only partial. As shown by the simultaneous identification of two polysaccharides in a novel biological context and of binding specificities of two CBMs and one SusD-like protein, the ‘double blind’ strategy that we describe here represents a powerful alternative to catch up on the lack of biochemical knowledge with the overwhelming wave of (meta)genomic data.

## Methods

### Collection and processing of algal material

Macroalgal samples were collected in their natural environments: *Porphyra umbilicalis* (Donegal, Ireland, GPS coordinates: 54.63, −8.14), *Ascophyllum nodosum* (Grenaa, Denmark, 56.42, 10.88), *Palmaria palmata* (Odder, Denmark, 55.97, 10.25), *Himanthalia elongata* (France, Plouguerneau, 48.62, −4.56), *Laminaria digitata*, *Fucus vesiculosus*, *Chondrus crispus*, *Ulva* sp. (France, Roscoff, 48.72, −3.98) and *Gracilaria verrucosa* (Lüderitz, Namibia, −26.65, 15.14). These samples were washed with water and their epiphytes eliminated. *Chlamydomonas reinhardtii*, *Arabidopsis thaliana* and *Brachypodium distachyon* were cultivated in Copenhagen University (Denmark).

### Preparation of marine glycoarrays

All algal and plant samples were freeze-dried, finely ground and processed to obtain their alcohol insoluble residue (AIR) as described previously^47^. Cell wall polysaccharides were sequentially extracted using procedures adapted to the material under study. Brown algal samples were treated with 2% CaCl_2_ at 80 °C, 0.2 M HCl, 3% Na_2_CO_3_, 50 mM CDTA and 4 M NaOH with 1% v/v NaBH_4_. Red algal samples used 0.2 M KCl, water 80 °C, CDTA and NaOH. Samples from green algae/plants were extracted with CDTA and NaOH. Purified polysaccharides were sourced as follows: arabinan, ivory nut mannan, tamarind seed xyloglucan, Icelandic moss lichenan, carboxymethyl cellulose from Megazyme; birchwood xylan, laminarin, fucoidan F5631, κ-, ι- and λ- carrageenans from Sigma; three sodium alginates (manuronic/guluronic ratios: 0.5, 0.9 and 2.1) from DuPont, bacteriological agar from Scharlau, agarose from SeaKem and Ulvan polysaccharides from Oligotech. The porphyran was prepared as described previously^32^. All polysaccharides were dissolved in deionised water, except mannan which was dissolved in 4 M NaOH. All these samples were used to prepare the glycoarray as described^21^, and printed onto a nitrocellulose membrane with a piezoelectric Sprint microarrayer (Arrayjet, Roslin, UK). The sample carrier used was printing buffer (55.2% glycerol, 44% deionised water, 0.8% Triton X-100). Each sample was printed four times followed by a 4-fold dilution (starting at 1 mg/ml for defined polysaccharides and 5 mg/mL for algal and plant material), giving a final count of 16 spots per sample on the array.

### Medium scale expression of recombinant proteins

Twenty four target genes encoding putative carbohydrate-binding proteins (10 CBMs, 14 SusD-like proteins, Supplementary Table 1) were selected from the genome sequence of the marine bacterium *Zobellia galactanivorans*. The genes were cloned and heterologously expressed using a medium throughput strategy as previously described^19^. In short, the open reading frames, trimmed to the SusD-like/CBM binding domains only, were amplified by PCR and using primers incorporating specific restriction sites compatible with our ligation strategies. The amplification products were cloned into the pFO4 and pGEX vectors encoding an N-terminal His_6_-tag and a GST-tag, respectively. Recombinant plasmids were used to transform *E. coli* strains DH5α. The validated plasmids were used to transform appropriate *E. coli* strains BL21(DE3) and BL21, respectively. Screening of protein expression was performed at a small-scale range using a 24-well plate format and the autoinducible ZYP5052 media with ampicillin. The plates were centrifuged at 1,200 g for 20 min at 4 °C and the pelleted bacteria were resuspended in a lysis buffer containing 50 mM Tris pH 8, 300 mM NaCl, 1 mg/mL lysozyme, 0.1 mg/mL DNAse and a tablet of protease inhibitor cocktail (Roche). The cell lysates were further centrifuged at 12,000 g for 20 min. The supernatants contained the soluble expressed fractions. The remaining cell pellets, which contained the insoluble expressed fractions, were extracted with 6 M urea. All fractions were analysed on SDS-PAGE (Supplementary Fig. S3). Protein concentrations of the soluble fractions were estimated by the Bradford method.

### Expression and purification of selected probes

The expression volume was scaled up for four selected probes. The corresponding BL21(DE3) and BL21 strains were grown at 37 °C overnight in Luria-Bertani (LB) medium containing ampicillin. The cultures were diluted 1:100 with auto-inducible ZYP5052 medium containing ampicillin and subjected to further incubation at 20 °C until the culture density reached saturation. After centrifugation at 5000 g for 10 min at 4 °C, pelleted bacteria were stored at −20 °C. For the His-tagged probes the cells were either resuspended in a buffer containing 25 mM HEPES pH7.5, 300 mM NaCl (SusD-213, SusD-3468) or a buffer containing 50 mM Tris pH8.0, 100 mM NaCl (CBM42). Both buffers were supplemented with 15 mM imidazole, a mixture of antiproteases and DNase. Bacteria were disrupted using a French press before centrifugation at 12,000 g for 90 min at 4 °C. Supernatants were applied onto a HisPrep column (GE Healthcare) charged with 100 mM NiSO_4_. After washing, the bound proteins were eluted with a linear gradient of imidazole ranging from 15 mM to 1 M. The proteins eluted were collected, concentrated with Ultrafiltration Cell (Millipore, Amicon) and further purified on a calibrated size exclusion chromatography column (Superdex, GE Healthcare). For the GST-tagged probe the cells were resuspended in PBS pH7.3 containing a mixture of anti-proteases and DNase. Bacteria were disrupted using a French press before centrifugation at 12000 g for 90 min at 4 °C. Supernatants were applied onto a Glutathione Sepharose High Performance column (GE Healthcare). After washing, the bound proteins were eluted with 50 mM Tris pH 8.0 containing 10 mM reduced glutathione. The proteins were further dialysed against a buffer containing 50 mM Tris pH 8.0, 100 mM NaCl. Final protein concentrations were estimated by the Bradford method.

### Glycoarray probing and analysis

The marine glycoarrays were first probed with the non-purified supernatants of cell lysates, similarly as described for purified proteins^23^. Briefly, the fractions were diluted at 30 µg/mL in 5% milk powder in PBS (MP/PBS) and applied to the arrays for 1 h, these arrays were then washed and immersed in a 5% MP/PBS dilution of anti-polyhistidine alkaline phosphate conjugate (Sigma) for his tagged proteins at 1:1000, or anti-glutathione-S-transferase (GST)–alkaline phosphatase conjugate (Sigma) for GST tagged proteins at 1:250. Finally, the arrays were developed using a mixture of 5-bromo-4-chloro-3-indolylphosphate (BCIP) and nitro blue tetrazolium (NBT) in alkaline phosphatase buffer (100 mM NaCl, 5 mM MgCl_2_, 100 mM diethanolamine, pH 9.5) scanned and quantified as described^23^ with an Array Pro-Analyzer 6.3 software (Media Cybernetics, USA) to be ultimately converted into heatmaps. Arrays treated with secondary antibodies only were used as untreated controls to subtract the background and obtain a net value for each measurement. A second round of glycoarray probing was performed using four selected purified proteins at 30 µg/mL, based on the procedure described above and compared with antibodies at 1:10 from Plant Probes (LM10, LM11, LM15, LM24, LM25).

### Biochemical assays

Additional polysaccharides were sourced as follows: oat spelt xylan, beechwood xylan and wheat arabinoxylan from Megazyme; ι- and λ-carrageenans from DuPont; carboxy methyl cellulose from Sigma; κ-carrageenan from Sanofi; agarose from Eurogentec and laminarin from Goëmar, France. The MLX from *P. palmata* was extracted using 0.5 M NaOH as described^27^. *C. crispus* algae were collected in Roscoff and the gametophytes and sporophytes sorted using the acetal-resorcinol assay^48^. The algae were air-dried, finely ground and resuspended in water (80 °C, 4 hours) before centrifugation at 1500 g for 30 min. Supernatants were precipitated with 5 volumes and ethanol and air-dried before use. Microtitre plates (Maxisorb, Thermo Scientific) were coated overnight at 4 °C with 50 μg/mL of the appropriate polysaccharide in PBS. Unbound polysaccharides were washed out using tap water, and all binding sites on the plates were blocked with a solution of 5% MP/PBS. The plates were rinsed in tap water and the purified probes were applied as serial dilutions in MP/PBS. After 1 hour at room temperature (RT), the plates were washed extensively and a 1000-fold dilution of the corresponding secondary antibody linked to HRP was applied in MP/PBS (e.g. anti-His HRP and anti-GST HRP, both from Sigma). After 1 hour at RT the plates were washed and developed with the HRP substrate (0.1 M sodium acetate buffer, pH 6.0, 1% tetramethyl benzidine, 0.006% (v/v) H_2_O_2_). When complete (usually in the 15–20 min range), the reaction was stopped with 2.5 M H_2_SO_4_ and absorbance was read at 450 nm. For each probe to be screened, all putative ligands were assayed in parallel.

## Electronic supplementary material

Supplementary Figures S1-S5

Supplementary Table S1

## References

[1] Popper ZA (2011). Evolution and diversity of plant cell walls: from algae to flowering plants. Annual Review of Plant Biology.

[2] Hehemann J-H, Boraston AB, Czjzek M (2014). A sweet new wave: structures and mechanisms of enzymes that digest polysaccharides from marine algae. Current Opinion in Structural Biology.

[3] Lombard V, Golaconda Ramulu H, Drula E, Coutinho PM, Henrissat B (2014). The carbohydrate-active enzymes database (CAZy) in 2013. Nucleic Acids Research.

[4] Deniaud-Bouët E, Hardouin K, Potin P, Kloareg B, Hervé C (2017). A review about brown algal cell walls and fucose-containing sulfated polysaccharides: Cell wall context, biomedical properties and key research challenges. Carbohydrate polymers.

[5] Lechat H, Amat M, Mazoyer J, Buléon A, Lahaye M (2000). Structure and distribution of glucomannan and sulfated glucan in the cell walls of the red alga *Kappaphycus alvaezii* (Gigartinales, Rhodophyta). Journal of Phycology.

[6] Salmeán AA (2017). Insoluble (1 → 3), (1 → 4)-β-D-glucan is a component of cell walls in brown algae (Phaeophyceae) and is masked by alginates in tissues. Scientific Reports.

[7] Azam F, Malfatti F (2007). Microbial structuring of marine ecosystems. Nat Rev Micro.

[8] Buchan A, LeCleir GR, Gulvik CA, Gonzalez JM (2014). Master recyclers: features and functions of bacteria associated with phytoplankton blooms. Nat Rev Micro.

[9] Giovannoni SJ, Stingl U (2005). Molecular diversity and ecology of microbial plankton. Nature.

[10] Glockner FO (2003). Complete genome sequence of the marine planctomycete *Pirellula* sp. strain 1. Proc Natl Acad Sci USA.

[11] Ivars-Martinez E (2008). Comparative genomics of two ecotypes of the marine planktonic copiotroph *Alteromonas macleodii* suggests alternative lifestyles associated with different kinds of particulate organic matter. ISME J.

[12] Teeling H (2012). Substrate-controlled succession of marine bacterioplankton populations induced by a phytoplankton bloom. Science.

[13] Barbeyron T (2016). Habitat and taxon as driving forces of carbohydrate catabolism in marine heterotrophic bacteria: example of the model algae-associated bacterium *Zobellia galactanivorans* DsijT. Environmental Microbiology.

[14] Koropatkin NM, Martens EC, Gordon JI, Smith TJ (2008). Starch catabolism by a prominent human gut symbiont is directed by the recognition of amylose helices. Structure.

[15] Shipman JA, Berleman JE, Salyers AA (2000). Characterization of four outer membrane proteins involved in binding starch to the cell Surface of *Bacteroides thetaiotaomicron*. Journal of Bacteriology.

[16] Cho KH, Salyers AA (2001). Biochemical analysis of interactions between outer membrane proteins that contribute to starch utilization by *Bacteroides thetaiotaomicron*. Journal of Bacteriology.

[17] Cuskin F (2015). Human gut Bacteroidetes can utilize yeast mannan through a selfish mechanism. Nature.

[18] Larsbrink, J. *et al*. A discrete genetic locus confers xyloglucan metabolism in select human gut Bacteroidetes. *Nature* (2014). 10.1038/nature12907 PMC4282169 24463512

[19] Groisillier A (2010). MARINE-EXPRESS: taking advantage of high throughput cloning and expression strategies for the post-genomic analysis of marine organisms. Microb Cell Fact.

[20] Terwilliger TC (2004). Structures and technology for biologists. Nat Struct Mol Biol.

[21] Moller I (2007). High-throughput mapping of cell-wall polymers within and between plants using novel microarrays. The Plant Journal.

[22] Pedersen HL (2012). Versatile high resolution oligosaccharide microarrays for plant glycobiology and cell wall research. Journal of Biological Chemistry.

[23] Vidal-Melgosa S (2015). A new versatile microarray-based method for high throughput screening of carbohydrate-active enzymes. J Biol Chem.

[24] Jam M (2016). Unraveling the multivalent binding of a marine family 6 carbohydrate-binding module with its native laminarin ligand. FEBS J.

[25] Doublier JL, Garnier C, Renard D, Sanchez C (2000). Protein–polysaccharide interactions. Current Opinion in Colloid & Interface Science.

[26] Burton RA, Fincher GB (2009). (1,3;1,4)-beta-D-glucans in cell walls of the poaceae, lower plants, and fungi: a tale of two linkages. Mol Plant.

[27] Deniaud E, Quemener B, Fleurence J, Lahaye M (2003). Structural studies of the mix-linked beta-(1 → 3)/beta-(1 → 4)-D-xylans from the cell wall of *Palmaria palmata* (Rhodophyta). Int J Biol Macromol.

[28] Labourel A (2014). The β-Glucanase ZgLamA from *Zobellia galactanivorans* evolved a bent active site adapted for efficient degradation of algal laminarin. Journal of Biological Chemistry.

[29] McCartney L, Gilbert HJ, Bolam DN, Boraston AB, Knox JP (2004). Glycoside hydrolase carbohydrate-binding modules as molecular probes for the analysis of plant cell wall polymers. Analytical Biochemistry.

[30] McCartney L, Marcus SE, Knox JP (2005). Monoclonal antibodies to plant cell wall xylans and arabinoxylans. J. Histochem. Cytochem..

[31] Collén, J. *et al*. In *Advances in Botanical Research* Vol. 71 (ed Bourgougnon Nathalie), 53–89 (Academic Press, 2014).

[32] Correc G, Hehemann J-H, Czjzek M, Helbert W (2011). Structural analysis of the degradation products of porphyran digested by *Zobellia galactanivorans* β-porphyranase A. Carbohydrate polymers.

[33] Ficko-Blean E, Hervé C, Michel G (2015). Sweet and sour sugars from the sea: the biosynthesis and remodeling of sulfated cell wall polysaccharides from marine macroalgae. Perspectives in Phycology.

[34] Kloareg B, Quatrano RS (1988). Structure of the cell walls of marine algae and ecophysiological functions of the matrix polysaccharides. Oceanogr. Mar. Biol. Annu. Rev..

[35] Raimundo, S. C., Pattathil, S., Eberhard, S., Hahn, M. G. & Popper, Z. A. β-1,3-Glucans are components of brown seaweed (Phaeophyceae) cell walls. *Protoplasma*, 1–20 (2016). 10.1007/s00709-016-1007-6 27562783

[36] Bae B (2008). Molecular basis for the selectivity and specificity of ligand recognition by the family 16 carbohydrate-binding modules from *Thermoanaerobacterium polysaccharolyticum* ManA. Journal of Biological Chemistry.

[37] Miyanaga A (2004). Crystal structure of a family 54 α-l-arabinofuranosidase reveals a novel carbohydrate-binding module that can bind arabinose. Journal of Biological Chemistry.

[38] Labourel A. Structural and functional studies of enzymes involved in the laminarin metabolism of two emerging model organism, the brown alga *Ectocarpus siliculosus* and the marine bacterium *Zobellia galactanivorans*. PhD thesis, Université Pierre et Marie Curie, Paris (2013).

[39] Hervé C (2010). Carbohydrate-binding modules promote the enzymatic deconstruction of intact plant cell walls by targeting and proximity effects. Proc Natl Acad Sci USA.

[40] Bjursell MK, Martens EC, Gordon JI (2006). Functional genomic and metabolic studies of the adaptations of a prominent adult human gut symbiont, *Bacteroides thetaiotaomicron*, to the suckling period. Journal of Biological Chemistry.

[41] Bernalier-Donadille A (2010). Fermentative metabolism by the human gut microbiota. Gastroenterologie clinique et biologique.

[42] Flint HJ, Bayer EA, Rincon MT, Lamed R, White BA (2008). Polysaccharide utilization by gut bacteria: potential for new insights from genomic analysis. Nat Rev Micro.

[43] Mirande C (2010). Dietary fibre degradation and fermentation by two xylanolytic bacteria *Bacteroides xylanisolvens* XB1AT and *Roseburia intestinalis* XB6B4 from the human intestine. Journal of Applied Microbiology.

[44] Robert C, Del’Homme C, Bernalier-Donadille A (2001). Interspecies H2 transfer in cellulose degradation between fibrolytic bacteria and H2-utilizing microorganisms from the human colon. FEMS Microbiology Letters.

[45] Ficko-Blean E (2017). Carrageenan catabolism is encoded by a complex regulon in marine heterotrophic bacteria. Nature Communications.

[46] Mackenzie AK (2015). A Polysaccharide Utilization Locus from an uncultured Bacteroidetes phylotype suggests ecological adaptation and substrate versatility. Applied and Environmental Microbiology.

[47] Hervé C (2016). Arabinogalactan proteins have deep roots in eukaryotes: identification of genes and epitopes in brown algae and their role in *Fucus serratus* embryo development. New Phytologist.

[48] Yaphe W, Arsenault GP (1965). Improved resorcinol reagent for the determination of fructose, and of 3,6-anhydrogalactose in polysaccharides. Analytical Biochemistry.

