# Supplementary material for: Double blind microarray-based polysaccharide profiling enables parallel identification of uncharacterized polysaccharides and carbohydrate-binding proteins with unknown specificities
Source: Sci Rep. 2018 Feb 6;8:2500. doi: 10.1038/s41598-018-20605-9 (PMC5802718; doi:10.1038/s41598-018-20605-9)
Supplement: Supplementary file 1 — Supplementary Figures S1-S5 [file 41598_2018_20605_MOESM1_ESM.pdf]

Double blind microarray-based polysaccharide profiling enables parallel identification of uncharacterized polysaccharides and carbohydrate-binding proteins with unknown specificities

Armando A. Salmeán[1], Alexia Guillouzo[2], Delphine Duffieux[2], Murielle Jam[2], Maria Matard-Mann[2], Robert Larocque[2], Henriette L. Pedersen[1], Gurvan Michel[2], Mirjam Czjzek[2], William G.T. Willats[1,3,*] and Cécile Herve[2,*]

[1]*Department of Plant and Environmental Sciences, University of Copenhagen, Thorvaldsensvej 40, 1871 Frederiksberg, Denmark;* [2]*Sorbonne Universités, UPMC Univ Paris 06, CNRS, UMR 8227, Integrative Biology of Marine Models, Station Biologique de Roscoff, CS 90074, Roscoff, France;*

[3]*Present address: William G.T. Willats, Newcastle University, Newcastle upon Tyne, United Kingdom*

[*]*Correspondence: William G.T. Willats, william.willats@newcastle.ac.uk; Cécile Hervé, cecile.herve@sb-roscoff.fr*

			CBM									SusD							neg. ctrls		
			CBM6 (1)	CBM6 (2)	CBM6 (3)	CBM13	CBM16 (1)	CBM22	CBM35	CBM42	CBM47	SusD-213	SusD-2202	SusD-2218	SusD-3468	SusD-3508	SusD-3894	SusD-3946	B-PAB0034	*E. coli(pFO4)*	*E. coli*
BROWN ALGAE	$CaCl_2$	*Laminaria digitata*	17	40	13	0	0	0	0	0	37	11	7	26	22	7	10	7	26	0	21
		Fucus vesiculosus	16	24	18	20	18	19	21	19	20	20	19	20	22	19	20	20	18	21	15
		Ascophyllum nodosum	10	17	11	12	9	10	12	11	16	12	11	11	13	10	12	11	8	12	8
		Himanthalia elongata	8	20	11	12	9	8	12	11	14	13	11	12	14	10	11	11	11	11	9
	HCl	*Laminaria digitata*	0	0	0	0	0	0	0	0	0	0	0	0	0	0	0	0	18	0	17
		Fucus vesiculosus	0	12	0	0	0	0	0	0	12	0	0	6	5	0	0	0	0	0	0
		Ascophyllum nodosum	5	15	0	0	0	0	0	0	15	0	0	8	6	0	0	0	0	0	0
		Himanthalia elongata	0	0	0	0	0	0	0	0	0	0	0	0	0	0	0	0	0	0	0
	Na_2CO_3	*Laminaria digitata*	0	0	0	0	0	0	0	0	0	0	0	0	0	0	0	0	18	0	17
		Fucus vesiculosus	13	20	14	13	10	11	13	12	18	13	12	15	15	9	14	13	9	12	9
		Ascophyllum nodosum	18	29	18	14	11	13	14	13	26	15	14	22	19	12	18	14	10	13	10
		Himanthalia elongata	15	26	16	15	11	12	16	14	24	15	14	18	17	12	16	14	11	15	10
	CDTA	*Laminaria digitata*	0	0	0	0	0	0	0	0	0	0	0	0	0	0	0	0	13	0	12
		Fucus vesiculosus	0	9	0	6	0	0	6	0	6	5	0	0	7	0	0	0	0	0	0
		Ascophyllum nodosum	11	19	12	11	8	9	12	10	17	10	10	12	13	8	10	10	8	10	7
		Himanthalia elongata	0	8	6	0	0	0	0	0	6	0	0	0	6	0	0	5	0	0	0
	NaOH	*Laminaria digitata*	0	6	0	0	0	0	0	0	6	0	0	0	0	0	0	0	0	0	0
		Fucus vesiculosus	6	12	8	7	0	0	8	7	10	7	7	8	7	0	6	7	5	7	0
		Ascophyllum nodosum	11	21	13	10	6	8	11	9	20	10	9	14	13	7	10	10	7	10	6
		Himanthalia elongata	0	0	0	0	0	0	0	0	6	0	0	0	0	0	0	0	0	0	0
RED ALGAE	KCl	*Porphyra umbilicalis*	0	0	0	0	0	0	0	0	0	0	0	0	0	0	0	0	0	0	0
		Palmaria palmata	0	0	0	0	0	0	0	22	0	0	0	0	0	0	0	0	0	0	0
		Gracilaria verrucosa	0	0	0	0	0	0	0	0	0	0	0	0	0	0	0	0	0	0	0
		C. crispus (gametophyte)	6	11	0	0	0	0	0	0	14	0	0	7	6	0	0	0	0	0	0
		C. crispus (sporophyte)	18	44	11	0	0	0	0	0	39	0	0	32	17	0	0	0	5	0	0
	H_2O	*Porphyra umbilicalis*	0	0	0	0	0	0	0	0	0	0	0	0	0	0	0	0	0	0	0
		Palmaria palmata	0	0	0	0	0	0	0	10	0	0	0	0	0	0	0	0	0	0	0
		Gracilaria verrucosa	0	0	0	0	0	0	0	0	0	0	0	0	0	0	0	0	0	0	0
		C. crispus (gametophyte)	7	14	0	0	0	0	0	0	16	0	0	9	7	0	0	0	0	0	0
		C. crispus (sporophyte)	16	35	11	0	0	0	0	0	36	9	0	23	13	0	8	0	6	0	0
	CDTA	*Porphyra umbilicalis*	0	0	0	0	0	0	0	0	0	0	0	0	0	0	0	0	0	0	0
		Palmaria palmata	0	0	0	0	0	0	0	6	0	0	0	0	0	0	0	0	0	0	0
		Gracilaria verrucosa	0	0	0	0	0	0	0	0	0	0	0	0	0	0	0	0	0	0	0
		C. crispus (gametophyte)	14	31	7	0	0	0	0	0	26	5	0	22	15	0	0	0	0	0	0
		C. crispus (sporophyte)	22	48	11	0	0	0	0	0	44	9	6	31	17	0	7	0	0	0	0
	NaOH	*Porphyra umbilicalis*	0	0	0	0	0	0	0	0	0	0	0	0	0	0	0	0	0	0	0
		Palmaria palmata	0	0	0	0	0	0	0	21	0	0	0	0	0	0	0	0	0	0	0
		Gracilaria verrucosa	0	0	0	0	0	0	0	0	0	0	0	0	0	0	0	0	0	0	0
		C. crispus (gametophyte)	17	40	11	0	0	0	0	0	35	8	0	24	18	0	7	0	0	0	0
		C. crispus (sporophyte)	17	39	9	0	0	0	0	0	37	5	0	26	14	0	0	0	0	0	0
PLANTS /GREEN ALGAE	CDTA	*Ulva sp.*	0	0	0	0	0	0	0	0	0	0	0	0	0	0	0	0	0	0	0
		C. reinhardtii	0	0	0	0	0	0	0	0	0	0	0	0	0	0	0	0	0	0	0
		Arabidopsis thaliana	0	0	0	0	0	0	0	0	0	0	0	0	0	0	0	0	0	0	0
		B. distachyon (leaf)	0	0	0	0	0	0	0	0	0	0	0	0	0	0	0	0	0	0	0
		B. distachyon (stem)	0	0	0	0	0	0	0	0	0	0	0	0	0	0	0	0	0	0	0
	NaOH	*Ulva sp.*	0	0	0	0	0	0	0	5	0	0	0	0	0	0	0	0	0	0	0
		C. reinhardtii	0	8	0	0	0	0	0	0	8	0	0	0	0	0	0	0	0	0	0
		Arabidopsis thaliana	0	0	0	0	0	0	0	0	0	0	0	0	0	0	0	0	0	0	0
		B. distachyon (leaf)	21	0	0	0	0	0	0	13	0	0	0	0	0	0	0	0	0	0	0
		B. distachyon (stem)	21	0	0	0	0	0	0	32	0	0	0	0	0	0	0	0	0	0	0
POLYSACCHARIDES		Kappa-carrageenan	14	29	8	0	0	0	0	0	24	7	0	19	12	0	0	0	0	0	0
		Iota-carrageenan	23	48	14	0	0	0	0	0	44	11	6	32	18	5	10	8	6	0	0
		Lambda-carrageenan	16	39	9	0	0	0	0	0	41	5	0	25	13	0	5	0	8	0	0
		Porphyran	0	0	0	0	0	0	0	0	0	0	0	0	0	0	0	0	0	0	0
		Agar	0	0	0	0	0	0	0	0	0	0	0	0	0	0	0	0	5	0	0
		agarose	0	0	0	0	0	0	0	0	0	0	0	0	0	0	0	0	0	0	0
		alginate 0.5	0	0	0	0	0	0	0	0	0	0	0	0	0	0	0	0	0	0	0
		alginate 0.9	0	0	0	0	0	0	0	0	0	0	0	0	0	0	0	0	0	0	0
		alginate 2.1	0	0	0	0	0	0	0	0	0	0	0	0	0	0	0	0	0	0	0
		Fucoidan	0	0	0	0	0	0	0	0	0	0	0	0	0	0	0	0	5	0	0
		Ulvan	0	0	0	0	0	0	0	0	0	0	0	0	0	0	0	0	0	0	0
		Cellulose CM	0	0	0	0	0	0	0	0	0	0	0	0	0	0	0	0	0	0	0
		Mixed linkage glucan	47	0	0	0	0	0	0	0	0	0	0	0	0	0	0	0	0	0	0
		Xylan (birchwood)	0	0	0	0	0	0	0	0	0	0	0	0	0	0	0	0	0	0	0
		Xyloglucan (tamarind)	0	0	0	0	0	0	0	0	0	6	0	0	0	0	0	0	0	0	0
		Mannan (ivory nut)	0	7	0	0	0	0	0	0	9	0	0	0	0	0	0	0	0	0	0
		Arabinan (sugar beet)	0	0	0	0	0	0	0	8	0	0	0	0	0	0	0	0	0	0	0

Average values (n=3) for HIS tagged proteins

Values <5 are considered 0

0	10	20	30	40	50	100

			CBM									SusD							neg. ctrls		
			CBM6 (1)	CBM6 (2)	CBM6 (3)	CBM13	CBM16 (1)	CBM22	CBM35	CBM42	CBM47	SusD-213	SusD-2202	SusD-2218	SusD-3468	SusD-3508	SusD-3894	SusD-3946	B-PAB0034	*E. coli(pFO4)*	*E. coli*
BROWN ALGAE	$CaCl_2$	*Laminaria digitata*	21	28	17	0	0	0	0	0	27	10	6	17	19	7	18	12	24	0	27
		Fucus vesiculosus	9	15	9	7	9	13	9	4	14	4	6	7	12	8	14	10	16	8	15
		Ascophyllum nodosum	5	11	4	1	3	4	4	1	12	2	2	3	6	3	8	4	7	3	7
		Himanthalia elongata	8	10	4	4	8	7	2	5	7	4	4	5	6	1	10	4	10	4	8
	HCl	*Laminaria digitata*	0	0	0	0	0	0	0	0	0	0	0	0	0	0	0	0	31	0	29
		Fucus vesiculosus	0	14	0	0	0	0	0	0	12	0	0	7	9	0	0	0	0	0	0
		Ascophyllum nodosum	9	21	0	0	0	0	0	0	20	0	0	10	11	0	0	0	0	0	0
		Himanthalia elongata	0	0	0	0	0	0	0	0	0	0	0	0	0	0	0	0	0	0	0
	Na_2CO_3	*Laminaria digitata*	0	0	0	0	0	0	0	0	0	0	0	0	0	0	0	0	31	0	29
		Fucus vesiculosus	7	9	5	1	4	4	2	2	11	2	2	5	8	2	8	4	8	2	8
		Ascophyllum nodosum	11	15	8	3	4	4	3	1	18	3	2	9	11	3	11	4	9	6	9
		Himanthalia elongata	7	11	3	2	4	2	2	3	14	1	1	5	6	1	6	1	10	4	9
	CDTA	*Laminaria digitata*	0	0	0	0	0	0	0	0	0	0	0	0	0	0	0	0	22	0	20
		Fucus vesiculosus	0	4	0	1	0	0	1	0	6	0	0	0	2	0	0	0	0	0	0
		Ascophyllum nodosum	5	10	3	1	2	1	1	2	12	1	1	4	6	1	9	1	7	4	6
		Himanthalia elongata	0	3	0	0	0	0	0	0	6	0	0	0	0	0	0	0	0	0	0
	NaOH	*Laminaria digitata*	0	6	0	0	0	0	0	0	6	0	0	0	0	0	0	0	0	0	0
		Fucus vesiculosus	6	6	3	0	0	0	1	1	9	1	1	2	3	0	5	2	5	2	0
		Ascophyllum nodosum	6	14	6	1	5	3	2	1	16	2	1	6	7	2	5	3	6	4	6
		Himanthalia elongata	0	0	0	0	0	0	0	0	5	0	0	0	0	0	0	0	0	0	0
RED ALGAE	KCl	*Porphyra umbilicalis*	0	0	0	0	0	0	0	0	0	0	0	0	0	0	0	0	0	0	0
		Palmaria palmata	0	0	0	0	0	0	0	11	0	0	0	0	0	0	0	0	0	0	0
		Gracilaria verrucosa	0	0	0	0	0	0	0	0	0	0	0	0	0	0	0	0	0	0	0
		C. crispus (gametophyte)	6	10	0	0	0	0	0	0	9	0	0	8	11	0	0	0	0	0	0
		C. crispus (sporophyte)	17	23	5	0	0	0	0	0	15	0	0	15	5	0	0	0	5	0	0
	H_2O	*Porphyra umbilicalis*	0	0	0	0	0	0	0	0	0	0	0	0	0	0	0	0	0	0	0
		Palmaria palmata	0	0	0	0	0	0	0	9	0	0	0	0	0	0	0	0	0	0	0
		Gracilaria verrucosa	0	0	0	0	0	0	0	0	0	0	0	0	0	0	0	0	0	0	0
		C. crispus (gametophyte)	7	13	0	0	0	0	0	0	14	0	0	9	8	0	0	0	0	0	0
		C. crispus (sporophyte)	13	19	15	0	0	0	0	0	24	16	0	16	15	0	14	0	5	0	0
	CDTA	*Porphyra umbilicalis*	0	0	0	0	0	0	0	0	0	0	0	0	0	0	0	0	0	0	0
		Palmaria palmata	0	0	0	0	0	0	0	6	0	0	0	0	0	0	0	0	0	0	0
		Gracilaria verrucosa	0	0	0	0	0	0	0	0	0	0	0	0	0	0	0	0	0	0	0
		C. crispus (gametophyte)	15	17	13	0	0	0	0	0	15	9	0	12	13	0	0	0	0	0	0
		C. crispus (sporophyte)	13	26	11	0	0	0	0	0	21	15	6	16	9	0	12	0	0	0	0
	NaOH	*Porphyra umbilicalis*	0	0	0	0	0	0	0	0	0	0	0	0	0	0	0	0	0	0	0
		Palmaria palmata	0	0	0	0	0	0	0	16	0	0	0	0	0	0	0	0	0	0	0
		Gracilaria verrucosa	0	0	0	0	0	0	0	0	0	0	0	0	0	0	0	0	0	0	0
		C. crispus (gametophyte)	19	24	14	0	0	0	0	0	16	13	0	16	13	0	12	0	0	0	0
		C. crispus (sporophyte)	7	15	11	0	0	0	0	0	15	9	0	12	7	0	0	0	0	0	0
PLANTS /GREEN ALGAE	CDTA	*Ulva sp.*	0	0	0	0	0	0	0	0	0	0	0	0	0	0	0	0	0	0	0
		C. reinhardtii	0	0	0	0	0	0	0	0	0	0	0	0	0	0	0	0	0	0	0
		Arabidopsis thaliana	0	0	0	0	0	0	0	0	0	0	0	0	0	0	0	0	0	0	0
		B. distachyon (leaf)	0	0	0	0	0	0	0	0	0	0	0	0	0	0	0	0	0	0	0
		B. distachyon (stem)	0	0	0	0	0	0	0	0	0	0	0	0	0	0	0	0	0	0	0
	NaOH	*Ulva sp.*	0	0	0	0	0	0	0	6	0	0	0	0	0	0	0	0	0	0	0
		C. reinhardtii	0	8	0	0	0	0	0	0	9	0	0	0	0	0	0	0	0	0	0
		Arabidopsis thaliana	0	0	0	0	0	0	0	0	0	0	0	0	0	0	0	0	0	0	0
		B. distachyon (leaf)	21	0	0	0	0	0	0	7	0	0	0	0	0	0	0	0	0	0	0
		B. distachyon (stem)	19	0	0	0	0	0	0	16	0	0	0	0	0	0	0	0	0	0	0
POLYSACCHARIDES		Kappa-carrageenan	13	25	10	0	0	0	0	0	21	8	0	17	11	0	0	0	0	0	0
		Iota-carrageenan	13	29	6	0	0	0	0	0	19	3	6	16	5	5	7	2	6	0	0
		Lambda-carrageenan	14	19	11	0	0	0	0	0	19	9	0	12	6	0	9	0	2	0	0
		Porphyran	0	0	0	0	0	0	0	0	0	0	0	0	0	0	0	0	0	0	0
		Agar	0	0	0	0	0	0	0	0	0	0	0	0	0	0	0	0	9	0	0
		agarose	0	0	0	0	0	0	0	0	0	0	0	0	0	0	0	0	0	0	0
		alginate 0.5	0	0	0	0	0	0	0	0	0	0	0	0	0	0	0	0	0	0	0
		alginate 0.9	0	0	0	0	0	0	0	0	0	0	0	0	0	0	0	0	0	0	0
		alginate 2.1	0	0	0	0	0	0	0	0	0	0	0	0	0	0	0	0	0	0	0
		Fucoidan	0	0	0	0	0	0	0	0	0	0	0	0	0	0	0	0	9	0	0
		Ulvan	0	0	0	0	0	0	0	0	0	0	0	0	0	0	0	0	0	0	0
		Cellulose CM	0	0	0	0	0	0	0	0	0	0	0	0	0	0	0	0	0	0	0
		Mixed linkage glucan	23	0	0	0	0	0	0	0	0	0	0	0	0	0	0	0	0	0	0
		Xylan (birchwood)	0	0	0	0	0	0	0	0	0	0	0	0	0	0	0	0	0	0	0
		Xyloglucan (tamarind)	0	0	0	0	0	0	0	0	0	10	0	0	0	0	0	0	0	0	0
		Mannan (ivory nut)	0	8	0	0	0	0	0	0	3	0	0	0	0	0	0	0	0	0	0
		Arabinan (sugar beet)	0	0	0	0	0	0	0	9	0	0	0	0	0	0	0	0	0	0	0

Standard deviation (n=3) for HIS tagged proteins

Group	Extraction	Sample	CBM16 (2)	SusD-1029	SusD-199	SusD-228	SusD-2433	SusD-3345	SusD-3571	SusD-3606	B-PAB0034	*E. coli* (pGEX)	*E. coli*
			**CBM**	**SusD**							**neg. ctrls**		
BROWN ALGAE	$CaCl_2$	*Laminaria digitata*	34	13	20	11	21	21	13	10	14	25	0
		Fucus vesiculosus	11	0	0	0	5	0	5	0	0	6	0
		Ascophyllum nodosum	8	0	0	0	0	0	0	0	0	0	0
		Himanthalia elongata	10	0	5	0	0	0	0	0	0	6	0
	HCl	*Laminaria digitata*	0	0	0	0	0	0	0	0	0	0	0
		Fucus vesiculosus	9	0	0	0	0	0	0	0	0	0	0
		Ascophyllum nodosum	10	0	0	0	5	0	0	0	0	0	0
		Himanthalia elongata	0	0	0	0	0	0	0	0	0	0	0
	Na_2CO_3	*Laminaria digitata*	0	0	0	0	0	0	0	0	0	0	0
		Fucus vesiculosus	14	0	7	0	9	0	0	0	0	9	0
		Ascophyllum nodosum	20	5	10	0	11	8	7	0	5	14	0
		Himanthalia elongata	14	5	8	0	9	5	6	0	0	12	0
	CDTA	*Laminaria digitata*	9	0	0	0	0	0	0	0	0	5	0
		Fucus vesiculosus	0	0	0	0	0	0	0	0	0	0	0
		Ascophyllum nodosum	10	0	0	0	6	0	0	0	0	6	0
		Himanthalia elongata	0	0	0	0	0	0	0	0	0	0	0
	NaOH	*Laminaria digitata*	13	7	6	0	8	0	5	0	0	10	0
		Fucus vesiculosus	7	0	0	0	0	0	0	0	0	0	0
		Ascophyllum nodosum	14	0	0	0	9	0	0	0	0	8	0
		Himanthalia elongata	0	0	0	0	0	0	0	0	0	0	0
RED ALGAE	KCl	*Porphyra umbilicalis*	0	0	0	0	0	0	0	0	0	0	0
		Palmaria palmata	0	0	0	0	0	0	0	0	0	0	0
		Gracilaria verrucosa	0	0	0	0	0	0	0	0	0	0	0
		C. crispus (gametophyte)	51	38	9	7	27	5	27	10	7	13	0
		C. crispus (sporophyte)	23	13	17	10	13	16	14	6	14	23	0
	H_2O	*Porphyra umbilicalis*	0	0	0	0	0	0	0	0	0	0	0
		Palmaria palmata	0	0	0	0	0	0	0	0	0	0	0
		Gracilaria verrucosa	0	0	0	0	0	0	0	0	0	0	0
		C. crispus (gametophyte)	72	42	5	0	50	0	22	18	5	11	0
		C. crispus (sporophyte)	24	16	15	11	15	19	17	13	16	21	0
	CDTA	*Porphyra umbilicalis*	0	0	0	0	0	0	0	0	0	0	0
		Palmaria palmata	0	0	0	0	0	0	0	0	0	0	0
		Gracilaria verrucosa	0	0	0	0	0	0	0	0	0	0	0
		C. crispus (gametophyte)	85	53	14	9	51	15	32	22	13	19	0
		C. crispus (sporophyte)	30	13	20	13	19	24	15	12	20	28	0
	NaOH	*Porphyra umbilicalis*	0	0	0	0	0	0	0	0	0	0	0
		Palmaria palmata	0	0	0	0	0	0	0	0	0	0	0
		Gracilaria verrucosa	0	0	0	0	0	0	0	0	0	0	0
		C. crispus (gametophyte)	74	48	15	7	44	18	28	19	15	23	0
		C. crispus (sporophyte)	23	12	15	9	13	16	13	7	13	21	0
PLANTS /GREEN ALGAE	CDTA	*Ulva sp.*	0	0	0	0	0	0	0	0	0	0	0
		C. reinhardtii	0	0	0	0	0	0	0	0	0	0	0
		Arabidopsis thaliana	0	0	0	0	0	0	0	0	0	0	0
		B. distachyon (leaf)	0	0	0	0	0	0	0	0	0	0	0
		B. distachyon (stem)	0	0	0	0	0	0	0	0	0	0	0
	NaOH	*Ulva sp.*	0	0	0	0	0	0	0	0	0	0	0
		C. reinhardtii	5	0	0	0	0	0	0	0	0	0	0
		Arabidopsis thaliana	0	0	0	0	0	0	0	0	0	0	0
		B. distachyon (leaf)	0	0	0	0	0	0	0	0	0	0	0
		B. distachyon (stem)	0	0	0	0	0	0	0	0	0	0	0
POLYSACCHARIDES		Kappa-carrageenan	52	25	7	0	43	10	9	14	9	11	0
		Iota-carrageenan	35	22	21	11	21	18	18	9	14	26	0
		Lambda-carrageenan	36	27	15	8	26	16	18	10	12	19	0
		Porphyran	0	0	0	0	0	0	0	0	0	0	0
		Agar	0	0	0	0	0	0	0	0	0	0	0
		agarose	0	0	0	0	0	0	0	0	0	0	0
		alginate 0.5	0	0	0	0	0	0	0	0	0	0	0
		alginate 0.9	0	0	0	0	0	0	0	0	0	0	0
		alginate 2.1	7	0	0	0	0	0	0	0	0	0	0
		Fucoidan	0	0	0	0	0	0	0	0	0	0	0
		Ulvan	0	0	0	0	0	0	0	0	0	0	0
		Cellulose CM	0	0	0	0	0	0	0	0	0	0	0
		Mixed linkage glucan	0	0	0	0	0	0	0	0	0	0	0
		Xylan (birchwood)	0	0	0	0	0	0	0	0	0	0	0
		Xyloglucan (tamarind)	0	0	0	0	0	0	0	0	0	0	0
		Mannan (ivory nut)	0	0	0	0	0	0	0	0	0	5	0
		Arabinan (sugar beet)	0	0	0	0	0	0	0	0	0	0	0

Average values (n=3) for GST tagged proteins

Values <5 are considered 0

0 | 20 | 30 | 40 | 50 | 100

			CBM	SusD							neg. ctrls		
			CBM16 (2)	SusD-1029	SusD-199	SusD-228	SusD-2433	SusD-3345	SusD-3571	SusD-3606	B-PAB0034	*E. coli(pGEX)*	*E. coli*
BROWN ALGAE	$CaCl_2$	*Laminaria digitata*	27	12	8	6	25	16	11	17	10	11	0
		Fucus vesiculosus	15	0	0	0	9	0	5	0	0	5	0
		Ascophyllum nodosum	14	0	0	0	0	0	0	0	0	0	0
		Himanthalia elongata	13	0	5	0	0	0	0	0	0	6	0
	HCl	*Laminaria digitata*	0	0	0	0	0	0	0	0	0	0	0
		Fucus vesiculosus	11	0	0	0	0	0	0	0	0	0	0
		Ascophyllum nodosum	17	0	0	0	9	0	0	0	0	0	0
		Himanthalia elongata	0	0	0	0	0	0	0	0	0	0	0
	Na_2CO_3	*Laminaria digitata*	0	0	0	0	0	0	0	0	0	0	0
		Fucus vesiculosus	14	0	2	0	9	0	0	0	0	8	0
		Ascophyllum nodosum	21	5	4	0	10	8	6	0	9	7	0
		Himanthalia elongata	16	5	3	0	11	9	5	0	0	6	0
	CDTA	*Laminaria digitata*	8	0	0	0	0	0	0	0	0	5	0
		Fucus vesiculosus	0	0	0	0	0	0	0	0	0	0	0
		Ascophyllum nodosum	12	0	0	0	7	0	0	0	0	5	0
		Himanthalia elongata	0	0	0	0	0	0	0	0	0	0	0
	NaOH	*Laminaria digitata*	12	7	0	0	7	0	9	0	0	3	0
		Fucus vesiculosus	6	0	0	0	0	0	0	0	0	0	0
		Ascophyllum nodosum	15	0	0	0	9	0	0	0	0	7	0
		Himanthalia elongata	0	0	0	0	0	0	0	0	0	0	0
RED ALGAE	KCl	*Porphyra umbilicalis*	0	0	0	0	0	0	0	0	0	0	0
		Palmaria palmata	0	0	0	0	0	0	0	0	0	0	0
		Gracilaria verrucosa	0	0	0	0	0	0	0	0	0	0	0
		C. crispus (gametophyte)	7	45	3	6	24	9	40	18	2	4	0
		C. crispus (sporophyte)	6	16	5	5	11	17	16	10	5	15	0
	H_2O	*Porphyra umbilicalis*	0	0	0	0	0	0	0	0	0	0	0
		Palmaria palmata	0	0	0	0	0	0	0	0	0	0	0
		Gracilaria verrucosa	0	0	0	0	0	0	0	0	0	0	0
		C. crispus (gametophyte)	30	38	5	0	43	0	33	31	5	5	0
		C. crispus (sporophyte)	24	14	14	10	26	16	15	22	16	14	0
	CDTA	*Porphyra umbilicalis*	0	0	0	0	0	0	0	0	0	0	0
		Palmaria palmata	0	0	0	0	0	0	0	0	0	0	0
		Gracilaria verrucosa	0	0	0	0	0	0	0	0	0	0	0
		C. crispus (gametophyte)	15	50	5	7	44	10	41	38	6	6	0
		C. crispus (sporophyte)	18	12	4	5	22	14	14	21	11	12	0
	NaOH	*Porphyra umbilicalis*	0	0	0	0	0	0	0	0	0	0	0
		Palmaria palmata	0	0	0	0	0	0	0	0	0	0	0
		Gracilaria verrucosa	0	0	0	0	0	0	0	0	0	0	0
		C. crispus (gametophyte)	22	44	5	7	40	11	38	34	6	7	0
		C. crispus (sporophyte)	12	13	4	8	15	6	15	12	6	5	0
PLANTS /GREEN ALGAE	CDTA	*Ulva sp.*	0	0	0	0	0	0	0	0	0	0	0
		C. reinhardtii	0	0	0	0	0	0	0	0	0	0	0
		Arabidopsis thaliana	0	0	0	0	0	0	0	0	0	0	0
		B. distachyon (leaf)	0	0	0	0	0	0	0	0	0	0	0
		B. distachyon (stem)	0	0	0	0	0	0	0	0	0	0	0
	NaOH	*Ulva sp.*	0	0	0	0	0	0	0	0	0	0	0
		C. reinhardtii	6	0	0	0	0	0	0	0	0	0	0
		Arabidopsis thaliana	0	0	0	0	0	0	0	0	0	0	0
		B. distachyon (leaf)	0	0	0	0	0	0	0	0	0	0	0
		B. distachyon (stem)	0	0	0	0	0	0	0	0	0	0	0
POLYSACCHARIDES		Kappa-carrageenan	46	23	6	0	38	9	9	23	8	10	0
		Iota-carrageenan	8	25	10	8	19	6	25	11	2	16	0
		Lambda-carrageenan	9	28	7	8	23	9	25	17	2	4	0
		Porphyran	0	0	0	0	0	0	0	0	0	0	0
		Agar	0	0	0	0	0	0	0	0	0	0	0
		agarose	0	0	0	0	0	0	0	0	0	0	0
		alginate 0.5	0	0	0	0	0	0	0	0	0	0	0
		alginate 0.9	0	0	0	0	0	0	0	0	0	0	0
		alginate 2.1	13	0	0	0	0	0	0	0	0	0	0
		Fucoidan	0	0	0	0	0	0	0	0	0	0	0
		Ulvan	0	0	0	0	0	0	0	0	0	0	0
		Cellulose CM	0	0	0	0	0	0	0	0	0	0	0
		Mixed linkage glucan	0	0	0	0	0	0	0	0	0	0	0
		Xylan (birchwood)	0	0	0	0	0	0	0	0	0	0	0
		Xyloglucan (tamarind)	0	0	0	0	0	0	0	0	0	0	0
		Mannan (ivory nut)	0	0	0	0	0	0	0	0	0	5	0
		Arabinan (sugar beet)	0	0	0	0	0	0	0	0	0	0	0

Standard deviation (n=3) for GST tagged proteins

Supplementary Figure S1. Full data set for the DB-CoMPP analysis of the marine samples. CoMPP binding profiles of the bacterial supernatants expressing proteins towards an extensive range of cell wall extracts and commercial polysaccharides. Results are means of three individual experiments replicates after subtraction of the corresponding negative controls. Both mean and SD values are shown for his- and GST-tagged probes. The colour scale in relation to absorbance values is shown. The highest mean signal value in the entire data set was set to 100 and all other signals adjusted accordingly. Values < 5 were considered as background and discarded. The main outcomes are summarized in Fig. 3 of the main manuscript.

HIS

		Replicate 1											
		Negative controls			Pre-subtraction			Post-subtraction			Normalized		
		B-PAB0034	*E. coli* (pFO4)	*E. coli*	SusD-2202	SusD-3508	CBM42	SusD-2202	SusD-3508	CBM42	SusD-2202	SusD-3508	CBM42
Red algae NaOH	*Palmaria palmata*	0	0	0	0	0	6	0	0	6	0	0	8
	C. crispus (gametophyte)	0	0	0	6	0	0	6	0	0	8	0	0
	C. crispus (sporophyte)	0	0	0	8	0	0	8	0	0	10	0	0
Polysaccharides	kappa-carrageenan	5	0	0	8	0	0	3	-5	-5	0	0	0
	iota-carrageenan	0	0	0	12	10	0	12	10	0	15	13	0
	lambda-carrageenan	10	0	11	7	0	0	-3	-10	-10	0	0	0
	xyloglucan (tamarind)	0	0	0	0	0	0	0	0	0	0	0	0

		Replicate 2											
		Negative controls			Pre-subtraction			Post-subtraction			Normalized		
		B-PAB0034	*E. coli* (pFO4)	*E. coli*	SusD-2202	SusD-3508	CBM42	SusD-2202	SusD-3508	CBM42	SusD-2202	SusD-3508	CBM42
Red algae NaOH	*Palmaria palmata*	0	0	0	0	0	20	0	0	20	0	0	77
	C. crispus (gametophyte)	7	0	0	0	0	0	-7	-7	-7	0	0	0
	C. crispus (sporophyte)	6	0	0	0	0	0	-6	-6	-6	0	0	0
Polysaccharides	kappa-carrageenan	0	0	0	0	0	0	0	0	0	0	0	0
	iota-carrageenan	12	0	0	5	0	8	-6	-12	-4	0	0	0
	lambda-carrageenan	8	0	0	0	0	0	-8	-8	-8	0	0	0
	xyloglucan (tamarind)	0	0	0	0	0	0	0	0	0	0	0	0

		Replicate 3											
		Negative controls			Pre-subtraction			Post-subtraction			Normalized		
		B-PAB0034	*E. coli* (pFO4)	*E. coli*	SusD-2202	SusD-3508	CBM42	SusD-2202	SusD-3508	CBM42	SusD-2202	SusD-3508	CBM42
Red algae NaOH	*Palmaria palmata*	0	0	0	0	0	37	0	0	37	0	0	52
	C. crispus (gametophyte)	0	0	0	8	7	9	8	7	9	11	9	12
	C. crispus (sporophyte)	0	0	0	0	0	0	0	0	0	0	0	0
Polysaccharides	kappa-carrageenan	5	0	0	6	8	7	1	3	2	0	0	0
	iota-carrageenan	0	0	0	0	5	0	0	5	0	0	7	0
	lambda-carrageenan	10	0	11	0	0	5	-10	-10	-5	0	0	0
	xyloglucan (tamarind)	0	0	0	0	0	7	0	0	7	0	0	10

		Average		
		Normalized		
		SusD-2202	SusD-3508	CBM42
Red algae NaOH	*Palmaria palmata*	0	0	45
	C. crispus (gametophyte)	6	0	0
	C. crispus (sporophyte)	0	0	0
Polysaccharides	kappa-carrageenan	0	0	0
	iota-carrageenan	0	7	0
	lambda-carrageenan	0	0	0
	xyloglucan (tamarind)	0	0	0

GST

		Replicate 1											
		Negative controls			Pre-subtraction			Post-subtraction			Normalized		
		B-PAB0034	*E. coli* (pGEX)	*E. coli*	SusD-228	SusD-3345	CBM16 (2)	SusD-228	SusD-3345	CBM16 (2)	SusD-228	SusD-3345	CBM16 (2)
Red algae NaOH	*Palmaria palmata*	0	0	0	0	0	0	0	0	0	0	0	0
	C. crispus (gametophyte)	14	22	0	0	15	80	-14	2	67	0	0	72
	C. crispus (sporophyte)	11	24	0	0	17	18	-11	6	7	0	7	8
Polysaccharides	kappa-carrageenan	13	20	0	0	13	86	-13	0	73	0	0	78
	iota-carrageenan	15	44	0	8	26	43	-7	11	28	0	12	31
	lambda-carrageenan	13	24	0	0	25	40	-13	13	27	0	14	29
	xyloglucan (tamarind)	0	0	0	0	0	0	0	0	0	0	0	0

		Replicate 2											
		Negative controls			Pre-subtraction			Post-subtraction			Normalized		
		B-PAB0034	*E. coli* (pGEX)	*E. coli*	SusD-228	SusD-3345	CBM16 (2)	SusD-228	SusD-3345	CBM16 (2)	SusD-228	SusD-3345	CBM16 (2)
Red algae NaOH	*Palmaria palmata*	0	0	0	0	0	0	0	0	0	0	0	0
	C. crispus (gametophyte)	10	16	0	14	10	49	5	0	39	5	0	43
	C. crispus (sporophyte)	9	15	0	15	10	14	7	1	6	8	0	6
Polysaccharides	kappa-carrageenan	0	0	0	0	0	0	0	0	0	0	0	0
	iota-carrageenan	11	23	0	20	14	27	9	3	15	10	0	17
	lambda-carrageenan	9	17	0	15	10	26	6	1	16	7	0	18
	xyloglucan (tamarind)	0	0	0	0	0	0	0	0	0	0	0	0

		Replicate 3											
		Negative controls			Pre-subtraction			Post-subtraction			Normalized		
		B-PAB0034	*E. coli* (pGEX)	*E. coli*	SusD-228	SusD-3345	CBM16 (2)	SusD-228	SusD-3345	CBM16 (2)	SusD-228	SusD-3345	CBM16 (2)
Red algae NaOH	*Palmaria palmata*	0	0	0	0	0	9	0	0	9	0	0	11
	C. crispus (gametophyte)	22	30	0	8	30	91	-14	8	70	0	10	87
	C. crispus (sporophyte)	21	25	0	12	21	37	-9	0	17	0	0	21
Polysaccharides	kappa-carrageenan	14	14	0	7	17	71	-7	3	57	0	0	72
	iota-carrageenan	15	12	0	6	15	35	-9	0	21	0	0	26
	lambda-carrageenan	14	17	0	7	11	43	-7	-3	29	0	0	37
	xyloglucan (tamarind)	0	0	0	0	0	0	0	0	0	0	0	0

		Average		
		Normalized		
		SusD-228	SusD-3345	CBM16 (2)
Red algae NaOH	*Palmaria palmata*	0	0	0
	C. crispus (gametophyte)	0	0	67
	C. crispus (sporophyte)	0	0	12
Polysaccharides	kappa-carrageenan	0	0	50
	iota-carrageenan	0	0	24
	lambda-carrageenan	0	0	28
	xyloglucan (tamarind)	0	0	0

Supplementary Figure S2. Example of the subtraction process performed as a key step for the DB-CoMPP analysis. Signals are shown for three his-tagged and three GST-tagged probes on a selection of polysaccharides, before and after subtraction of the signals obtained with the corresponding negative controls.

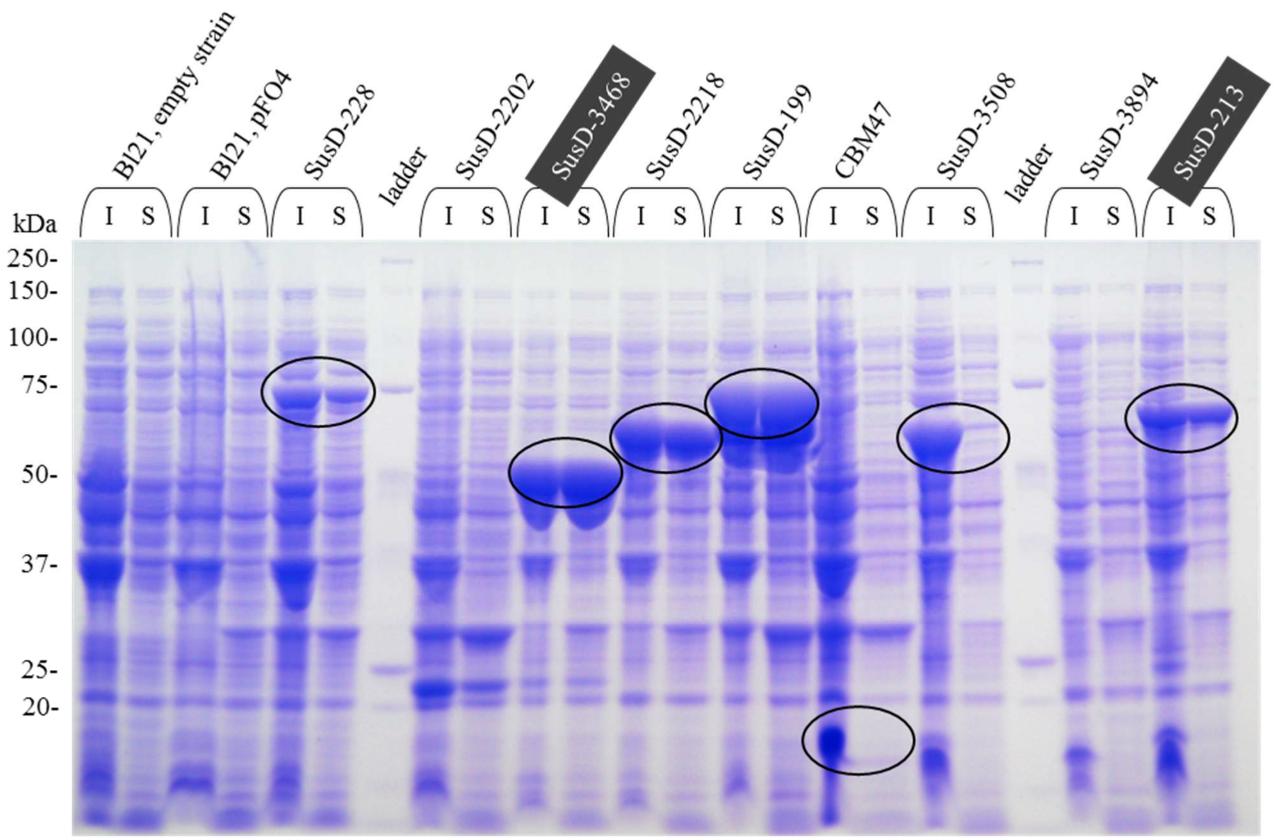

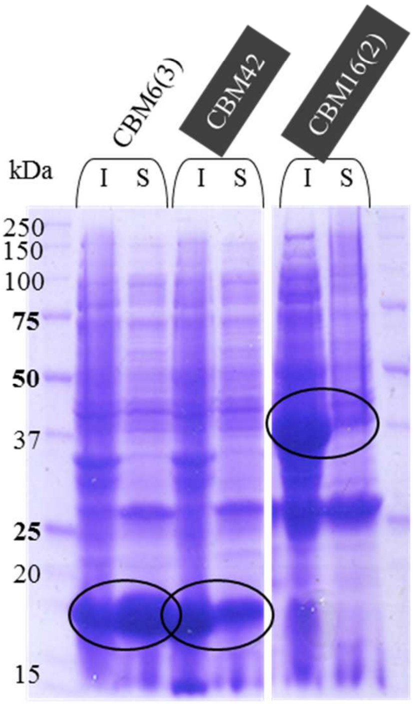

Supplementary Figure S3. SDS-PAGE of a selection of insoluble (I) and soluble (S) cellular extracts of the *E. coli* expression cultures. Black circles surround bands of correct size. A focus is made on the selected probes, namely CBM16(2), CBM42, SusD-213 and SusD-3468.

c

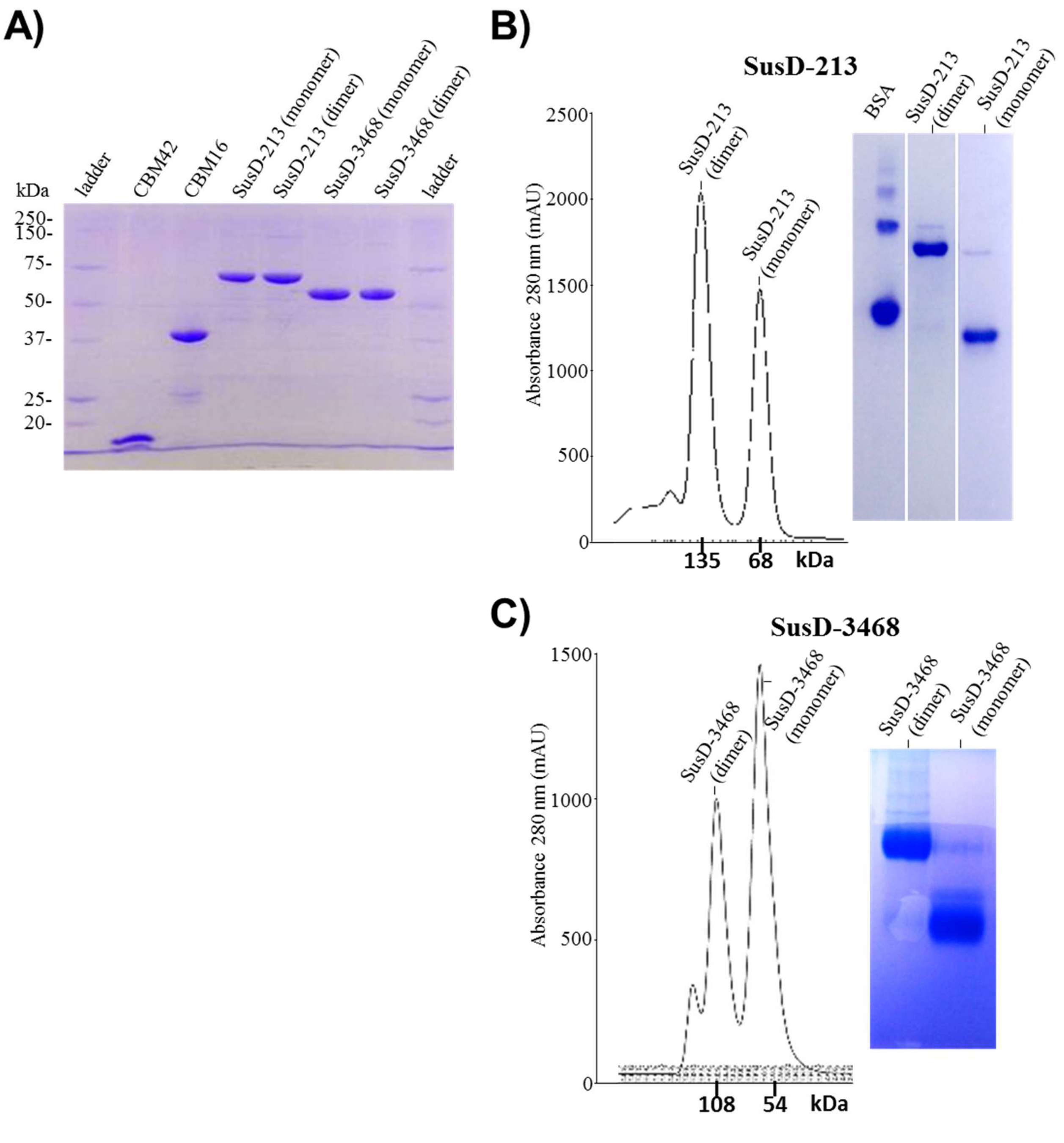

Supplementary Figure S4. Up-scale and purification of four selected probes. (a) SDS-PAGE of the four selected probes, namely CBM42, SusD-213, SusD-3468 (his-tagged) and CBM16 (GST-tagged). (b) Gel filtration of SusD-213 showing the occurrence of monomeric and dimeric forms. The corresponding native PAGE of the two forms is shown on the side. (c) Gel filtration of SusD-3468 showing the occurrence of monomeric and dimeric forms. The corresponding native PAGE of the two forms is shown on the side.

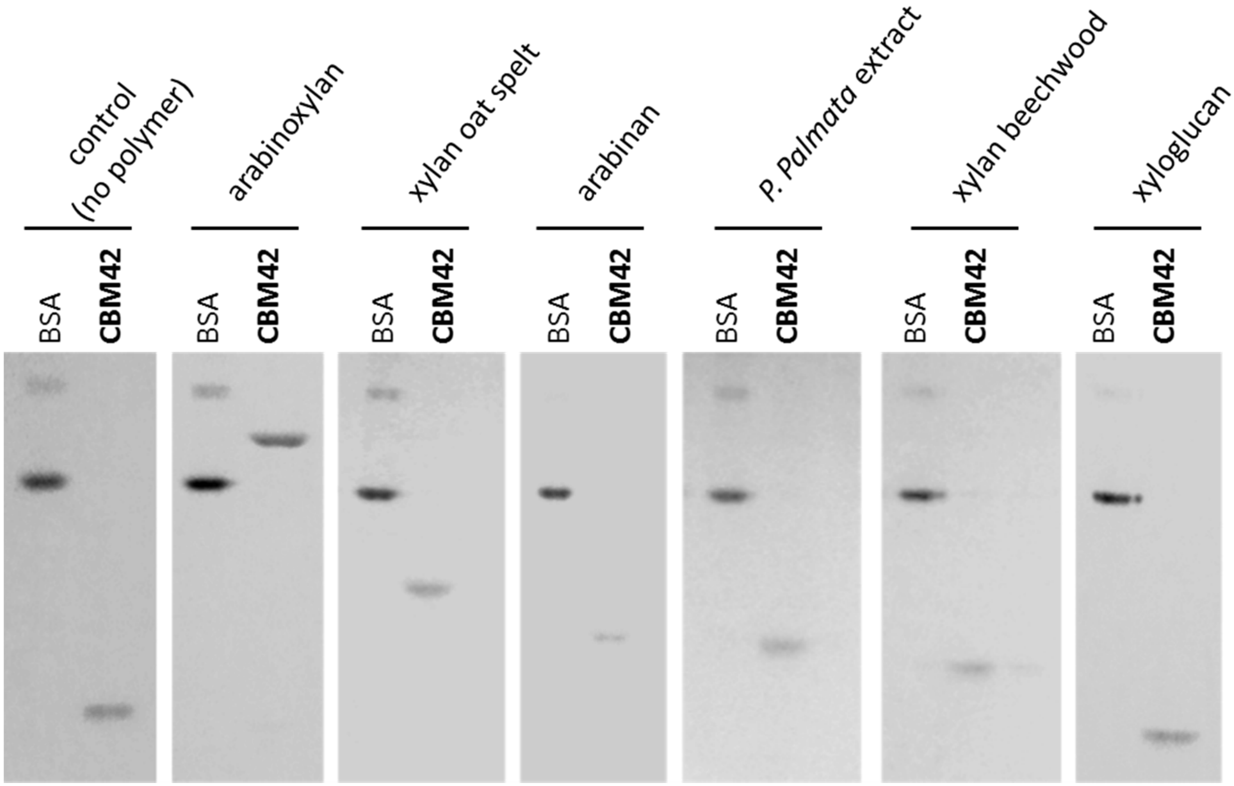

Supplementary Figure S5. Biochemical validation of the binding ability of CBM42 by AEG. The capacity of CBM42 to bind different polysaccharides was further evaluated by affinity gel electrophoresis. Polysaccharides were added at a final concentration of 0.1% before polymerization of 12% native acrylamide gels. 10 µl of CBM42 (0.6 mg/ml) were loaded on the gels. BSA was used as a negative control. Proteins were visualized by staining with Coomassie Blue. The progression of CBM42 into the gel was significantly delayed by arabinoxylan while no retardation was observed with xyloglucan. These results are in accordance with the result of the microtiter plate assay shown in Fig. 5 of the main manuscript.
